# Antioxidant Defense Mechanisms in Erythrocytes and in the Central Nervous System

**DOI:** 10.3390/antiox8020046

**Published:** 2019-02-18

**Authors:** Rafael Franco, Gemma Navarro, Eva Martínez-Pinilla

**Affiliations:** 1 Molecular Neurobiology Laboratory, Department of Biochemistry and Molecular Biomedicine, Biology School, University of Barcelona, Barcelona 08028, Spain; 2 Centro de Investigación en Red, Enfermedades Neurodegenerativas (CIBERNED), Instituto de Salud Carlos III, Madrid 28031, Spain; 3 Department of Biochemistry and Physiology, Pharmacy and Food Science School, University of Barcelona, Barcelona 08028, Spain; 4 Departamento de Morfología y Biología Celular, Facultad de Medicina, Universidad de Oviedo, Asturias 33006, Spain; 5 Instituto de Neurociencias del Principado de Asturias (INEUROPA), Asturias 33006, Spain; 6 Instituto de Salud del Principado de Asturias (ISPA), Asturias 33006, Spain

**Keywords:** CNS, fava beans, innate mechanisms, oxidative stress, pentose pathway

## Abstract

Differential antioxidant action is found upon comparison of organ/tissue systems in the human body. In erythrocytes (red blood cells), which transport oxygen and carbon dioxide through the circulatory system, the most important issue is to keep hemoglobin in a functional state that requires maintaining the haem group in ferrous (Fe^2+^) state. Conversion of oxidized Fe^3+^ back into Fe^2+^ in hemoglobin needs a special mechanism involving a tripeptide glutathione, glucose-6-phosphate dehydrogenase, and glucose and NADPH as suppliers of reducing power. Fava beans are probably a good resource to make the detox innate system more robust as the pro-oxidant molecules in this food likely induce the upregulation of members of such mechanisms. The central nervous system consumes more oxygen than the majority of human tissues, i.e., 20% of the body’s total oxygen consumption and, therefore, it is exposed to a high level of oxidative stress. This fact, together with the progressive age-related decline in the efficiency of the antioxidant defense system, leads to neuronal death and disease. The innate mechanism operating in the central nervous system is not well known and seems different to that of the erythrocytes. The strategies of antioxidant intervention in brain will be reviewed here.

## 1. The Paradoxical Issue of Oxygen-Based Life

Except in few organisms, life on Earth is based on the presence of oxygen in the atmosphere and in river and sea waters. Unless properly defined, antioxidant benefit is an oxymoron. Indeed, the action of antioxidants in an organism that depends on oxygen consumption is like adding a piece of blotting paper to decrease the sea level. As discussed elsewhere [1] the right use of an antioxidant molecule is to preserve degradation of dead matter. This is the reason why many processed foods include antioxidant to prolong the useful life of the product, i.e., to prevent food rotting.

From a chemical point of view, any antioxidant molecule has the property of being a reductant. In Earth’s life the most used (and powerful) reducing molecule is glucose that is oxidized by the glycolytic cycle in mammalian cells. Other compounds that are taken by mammals are fats, for instance those in sunflower or olive oils, whose reducing potential according to rigorous chemical rules is higher than that of glucose. In summary, taking chemical laws into account the intake of antioxidants at amount of 1 g per day should not have impact whatsoever in the degree of oxidation of cells in a mammal consuming a regular glucose and fat containing diet. We here use two mammalian body components, blood and central nervous cells, to review how they can be damaged by excess oxidation and what efficacious intervention might reduce oxidative exacerbation.

## 2. Sensitivity to Oxidation of Red Blood Cells

Red blood cells contain a substantial amount of hemoglobin that is used to transport oxygen and carbon dioxide to/from lungs. The haem group in this protein contains a ferrous (Fe^2+^) ion that coordinates with entering oxygen; when the ion is in ferric form (Fe^3+^) the protein is known as methemoglobin and is unable (or almost unable) to bind oxygen. This is one of the most known and fantastic paradoxes in the human body. The protein that captures oxygen and is in charge of oxygen transport has to maintain an ion in reduced form. Obviously, as many other oxidants, oxygen is able to convert Fe^2+^ into Fe^3+^, but if this occurs hemoglobin is not functional and the organism dies. 

To sustain life, mammalians must have a mechanism for impeding Fe^2+^ to Fe^3+^ conversion or for rapid conversion of an oxidized ion to its reduced form [2,3]. Oxidization of hemoglobin when oxygen is in the protein, is kinetically slow but it may happen. In this context, Evolution has developed a mechanism of detoxification that was deciphered with data taken from patients suffering from a monogenic disease. These patients present mutations in the glucose-6-phosphate dehydrogenase (G6PDH) gene that produce either little amounts of G6PDH, an enzyme that is enriched in red blood cells, or a protein with reduced enzyme activity. The clinical symptoms of patients with enzyme deficit appear, for instance, after ingestion of oxidants. The consequence is an increase in the levels of free radicals that leads to the damage of red blood cells. Symptoms depend on the degree of residual enzymatic activity; there are reported more than 300 mutations that lead to different clinical outcomes, from totally silent to the cases that are of interest here, namely those that produce hemolytic anemia upon infection or upon consumption of certain oxidant foods or drugs. In these cases, there is a decrease in erythrocyte counts by membrane disruption and a release of cell content. This prematurely destruction of red blood cells is known as hemolysis [4].

Interestingly, the episodes of hemolytic anemia in those patients helped to decipher the innate anti-oxidant mechanism of the erythrocyte. In non-pathological conditions, red blood cell G6PDH coverts the reducing power of glucose into reduced NADPH (reduced form of nicotine-adenine-dinucleotide) that in turn may serve to overcome oxidative stress. NADPH is produced mainly in a metabolic route known as the “pentose pathway” or the “pentose pathway shunt”, and uses the same fuel than glycolysis: glucose. Noteworthy, NADPH does not directly participate in the reduction of Fe^3+^ to Fe^2+^ in hemoglobin but it is the ultimate responsible of providing the reducing power needed for such reaction thus avoiding oxidation and denaturation of the protein [5,6,7]. In humans, this process seems to be mediated by the cytochrome B5 reductase, a NADH-dependent enzyme that catalyzes the conversion between methemoglobin and hemoglobin and whose congenital deficit leads to methemoglobinemia [8,9]. Production of NADPH is more dependent on the mentioned enzyme in red blood cells than in other tissues and this is the reason why the disease, due to mutation in the G6DPH gene, affects—almost exclusively—erythrocytes. 

**Figure 1:**
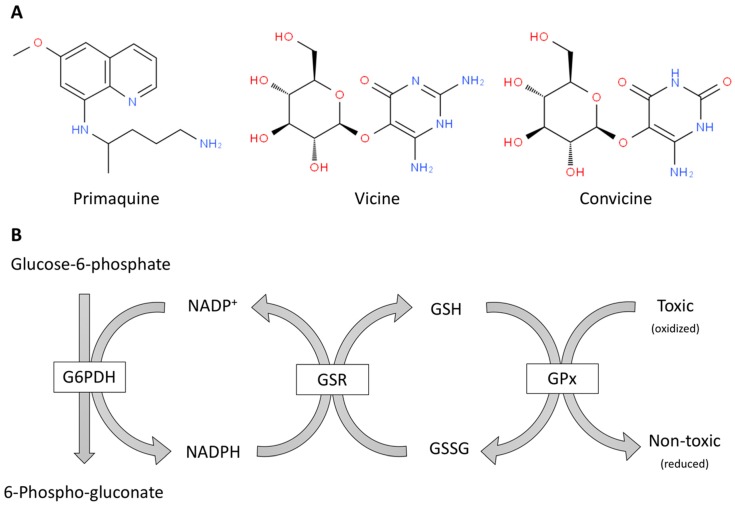
Oxidant molecules (**A**) and simplified scheme of antioxidant defense mechanisms mediated in erythrocytes by glutathione and NADPH (**B**). (**A**) Examples of pro-oxidant toxic molecules for red blood cells from G6PDH deficient patients: a drug, primaquine, and natural compounds from fava beans, vicine and convicine. (**B**) The enzymes are in boxes and the rest are substrates of these enzymes. G6PDH: Glucose-6-phosphate dehydrogenase, GPx: glutathione peroxidase; GSH: glutathione; GSR: glutathione-disulfide reductase; GSSG: glutathione disulfide.

The innate antioxidant mechanism in red blood cells (Figure 1) has been known for decades and is based on a tripeptide L-γ-glutamyl-L-cysteinyl-glycine, known as glutathione. Sulfhydryl is the most important group in this tripeptide; it may be in reduced form (SH) or in oxidized disulfide (S-S) form (see Figure 1). Reduction/oxidization cycles of glutathione are needed to restore the ferrous state of iron in hemoglobin. Remarkably, the restoration of reduced glutathione is fueled by NADPH (Figure 1). In summary, life is supported by oxidation, excess oxidation may occur in some situations and powerful innate mechanisms of detoxification have been evolved. Hemoglobin in erythrocytes, in this sense, is a paradigm of optimal design as requires reduced (ferrous) iron to be functional as oxygen carrier. The oxidative environment leads to production of ferric iron ion that is rapidly reduced by a glutathione-based mechanism. The whole process of detoxification in erythrocytes requires NADPH, whose reducing power ultimately comes from glucose. Then, mammalians use glucose as energy provider and as provider of reducing power in detox mechanisms. Fueled by glucose, glycolysis and the pentose pathway run in parallel in red blood cells.

Once the mechanism and its components (mediators/intermediates and source of reducing power) are known, one may wonder how to improve its efficacy, i.e., how to improve the antioxidant power of such mechanism. First of all, it must be stressed that the mechanism does not need anything else to be efficacious except in cases in which one of the components is altered (e.g., a G6PDH mutant with reduced activity) [2,3]. But assuming that one wants to make it more robust some interventions may be of interest. One is to take supplements of glutathione. Indeed, glutathione can be sold as a supplement and is easily available even in online “shops”. However, the intervention assumes that, upon oral administration, the unaltered tripeptide will reach the erythrocyte. This assumption is controversial and highly unlikely (discussed below) as peptidases are everywhere and chances are that the peptide will be degraded to its components before reaching its goal (see [10] for trial of efficacy to increase the levels of the compound). Therapeutic intervention in G6PDH deficiency depends on type and cause, but may require blood transfusion in acute hemolysis triggered by fava beans or pro-oxidant medication, bone marrow transplantation or, in rare cases, surgery for spleen removal. To our knowledge, glutathione is not “prescribed” as a treatment of choice in such circumstances. A note of caution concerns whether existing glutathione supplements should be commercialized in reduced or in oxidized form. As described elsewhere, intake of “antioxidant” by mouth may have local effects but not systemic effects as any reduced “antioxidant” will be quickly oxidized before arriving to blood and tissues [1]. The idea is that we may increase the concentration of a detox mechanism but it is fairly irrelevant whether it is orally administered in reduced or oxidized form. Supplementation directly using NADPH is also of little value for similar reasons. In a balanced diet, there is no shortage of nicotine amide dinucleotides (NAD/NADH or NADP/NADPH). It should be however noted that synthesis of these compounds in the human body requires a vitamin, niacin (nicotinic acid, a form of B3 vitamin), i.e., if any, supplementation with vitamin B3 may be considered whereas supplementation with NADPH is (quite likely) useless. Another related approach consists of increasing the activity of glutathione peroxidase (GPx); this is possible as it is an enzyme that depends on selenium [11]. In fact, an appropriate level of selenium (in diet or in supplements) is recommended. Then, it is likely that balanced nutrition fulfills the requirement of the red blood cell detox mechanism. 

Another strategy that is often forgotten also derives from the “work” of Evolution. The own body synthesizes the molecules needed to overcome a stressful situation, or be ready to do it when the stress occurs. Often, G6PDH deficiency is discovered when the patient eats fava beans. In fact, there are somehow isolated geographical regions in which the disease was prevalent and was known as “favism”. The solution was to avoid eating these vegetables. But this evidence gives rise to a straightforward assumption related with nutrition. Fava beans may be coaches for our blood cells meaning that, upon intake, the body reacts to decrease the oxidative stress due to some of the components of the food. Two related compounds, vicine and convicine, have been identified as the oxidative component(s) of the legume; they lead to significant higher hemolysis in red blood cells isolated from patients than in cells isolated from healthy controls [12,13]. Decades ago levodopa (L-DOPA; L-3,4-dihydroxyphenylalanine), a compound used for the treatment of Parkinson’s disease was suggested [14], but controversy arose and ultimate evidence was not possible unlike in the case of the oxidative components of anti-malarial medication (primaquine). Actually, some populations are diagnosed after prescription of primaquine in anti-malaria therapy. The prevalence of disease is higher in areas in which malaria is endemic and the hypothesis is that patients with malaria are more fertile or arrive to the age of reproduction before severe symptoms appear; it should be noted that malaria-endemic areas in Earth are also deficient in health conditions often accompanied by poor water quality and unbalanced nutrition. The first studies linking primaquine to alterations in erythrocyte membrane permeability and red blood cell fate are dated back in 1961 [15,16,17].

It is tempting to speculate that consumption by healthy humans of fresh or cooked fava bean legume makes the organism to up-regulate expression of the components of the detox machinery, G6PDH among them. This would be a mechanism designed by Evolution for which a clinical trial would be needed to correlate fava bean consumption with red blood cell G6PDH levels. Neither such information is available for mammalian animal models. This attractive hypothesis would be like the upregulation of muscle glycolytic enzymes upon exercise training. Indeed, the level of glucose-handling enzymes varies enormously from athletes to individuals with little physical activity. Furthermore, studies in exercise-trained rats show that diet affects muscle expression of G6PDH [18]. These mechanisms may be considered as hormetic, a word used more in the context of the biological effects of ionizing radiations [19]. Hormesis may lead to gene expression variation [20] and, interestingly, it has been reported that protection against hydrogen peroxide stress is afforded by superoxide radicals; in author’s own words: “*concentration-dependent roles of the superoxide radical comprise a form of hormesis and show one reactive oxygen species having a hormetic effect on the toxicity of another*” [21]. 

Although exceptions may occur, the increase in G6PDH activity seems beneficial in reducing the oxidative load, possibly because glutathione levels increase and, as far as red blood cells are concerned, it can keep hemoglobin at its functional state. Spironolactone is able to afford increases in G6PDH activity; the compound is a mineralocorticoid receptor blocker which is able to reduce oxidative stress in hypertensive and diabetic rats [22]. Supplementation with the natural product, α-lipoic acid (LA), in patients of G6PDH deficiency proves useful to balance the redox status. A further promising result of the same study is that the supplementation was able to increase the activity of the enzyme also in healthy controls [23]. In summary, to “help” the innate mechanisms seems beneficial and one of the successful ways to achieve it is by increasing the activity of G6PDH.

## 3. Sensitivity to Oxidation in the Central Nervous System

The most important cells in the central nervous system (CNS) are neurons and, accordingly, they must be preserved as much as possible from oxidative stress, which may cause neuronal dysfunction and/or death thus being a main contributor of neurological diseases. Recently, the susceptibility of CNS to oxidative stress has been reviewed and up to 13 possible causes have been provided; authors summarize them as it follows: *“unsaturated lipid enrichment, mitochondria, calcium, glutamate, modest antioxidant defense, redox active transition metals and neurotransmitter auto-oxidation. We review RNA oxidation as an underappreciated cause of oxidative stress”* [24]. Unfortunately, antioxidant defense mechanisms in the CNS are less known than in blood cells; a review on potential mechanism has been provided by [25]. The issue becomes more complex when one considers that, unlike red blood cells, neurons are very heterogeneous. In fact, there are dozens of neuronal types even in a given brain region. This complexity impacts on the knowledge of the neural innate detox networks/mechanisms and in developing strategies to prevent or control oxidative stress. We will review here some of the aspects that are seemingly relevant for CNS redox homeostasis and emit hypothesis on the approaches that would help CNS to minimize the impact of oxidative stress situations.

On the one hand, brain needs considerable amounts of oxygen for cell survival and for sustaining higher brain functions, so the innate detox mechanisms must be very powerful. On the other hand, even if the glutathione-based detox mechanism occurs in neural cells, the lack of neurological alterations in patients of G6PDH deficiency is of interest as it may be due to (i) low relevance of glutathione-based detox mechanisms or (ii) difficulties of primaquine or pro-oxidant fava bean molecules to cross the blood brain barrier. Irrespective of the real cause of lack of CNS affectation in patients, the strategy of eating fava beans to strengthen the innate mechanisms would not be appropriate for neurons. To our knowledge, there is no study addressing this issue since interventions seem difficult to implement in the CNS. Two of the above-mentioned molecules, spironolactone and LA, may be an option but we have not found information about their ability to enter into the brain. Spironolactone seems to enhance the antinociceptive action of opioids, thus suggesting that it may reach the brain but the concentrations used in the assays in laboratory animals were fairly high (100 mg/kg, i.p. administration) [26]. Also, intravenous administration of LA makes potentially possible that it reaches the brain and reduces oxidative stress after CNS damage, in a mechanism mediated by the nuclear factor erythroid 2-related factor 2 (Nrf-2) pathway [27] (see below). However, the antioxidant mechanism of LA is not well documented, something that likely reflects the lack of consensus on what are the innate mechanisms of addressing oxidative stress in the CNS. Therefore, it is urgent to decipher mechanisms to look for ways of potentiating them.

At present, we can look for information, guess potential mechanisms and consider basic chemical rules. In this sense, it is worth repeating that any antioxidant taken orally may not reach the CNS with intact “antioxidant” potential [1]. It is our opinion that oral antioxidants have negligible effect in the CNS. Antioxidant effect may eventually be reported in laboratory animals by direct administration of the antioxidant in the brain. Evidence suggests that instead of trying to reduce oxidative stress via administration of antioxidants, the human brain requires tools to enhance its own antioxidant capabilities.

**Figure 2:**
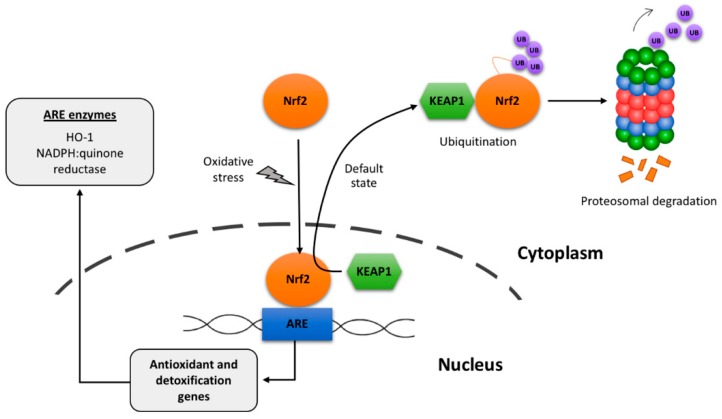
Scheme of default degradation and of oxidative-stress-induced activation of the transcription factor Nrf2. ARE: Antioxidant-response-element (components genes and proteins). It has been suggested that KEAP1/Nrf2 interaction occurs in the nucleus and then the complex is shuttled to the cytoplasm, however alternative mechanism may run in parallel (see [29]).

To end this review, we highlight a cell component in which scientists are placing hopes, among other, in cancer and in diseases of the CNS. As commented above, Nrf2 has antioxidant potential because it participates in the engagement of antioxidant pathways. It is a protein and its potential, as in the case of G6PDH, does not depend on itself but on the mechanism in which it participates. Even assuming that Nrf2 is a good clue, its role within the cell complicates identification of the actual detox mechanisms. Indeed, Nrf2 is a transcription factor (see [28] for review) and, therefore, it regulates the expression of some genes that code for “antioxidant enzymes”. According to chemical rules this nomenclature is misleading. Is G6PDH an antioxidant enzyme? Probably the answer is “no”, but in fact the enzyme is key in the innate mechanisms operating in red blood cells. One of the most referenced reviews indicates that: “*A major mechanism in the cellular defense against oxidative or electrophilic stress is activation of the Nrf2-antioxidant response element signaling pathway, which controls the expression of genes whose protein products are involved in the detoxication and elimination of reactive oxidants and electrophilic agents*” [29]. This sentence is quite appropriate as it correlates Nrf2 with the expression of proteins that participate in detox mechanisms. Let us look for the instrumental proteins and potential mechanisms. After a pro-oxidant situation/insult, Nrf2 becomes active by the mechanisms shown in Figure 2 and enters into the nucleus to exert its role as gene transcription regulator. The pathway, known as Nrf2/ARE, participates in regulating transcription of antioxidant response element (ARE) genes, i.e., genes that have an “ARE” regulatory element in the 5’ flanking region [30,31]. Nrf2 expression in human brain is moderate: 19 (relative units) *versus* 52 in thyroid or 92 in esophagus (www.ncbi.nlm.nih.gov/gene?cmd=Retrieve&dopt=full_report&list_uids=4780). Unlike the robust mechanism discovered in red blood cells, activation of the Nrf2 pathway requires an oxidative input that affects the interaction of proteins that regulate the transcriptional activity of Nrf2. Among the proteins that may interact with the transcription factor, Kelch-like ECH-associated protein 1 (KEAP1) contributes to ubiquitin-mediated degradation, thus impeding the induction of gene expression. One of the ways to activate the Nrf2-ARE pathway is to prevent the interaction with KEAP1, something that may be achieved, among other, by partner and localizer of BRCA2 (PALB2) [32] and p62 autophagy substrate [33]. 

On the one hand, it has been suggested that dietary phytochemicals may be activators of Nrf2 [34]. On the other hand, multiple studies have provided evidence of Nrf2-mediated upregulation of proteins that are somehow related to handling stress situations and, accordingly, that maintain REDOX homeostasis. To name a few, Nrf2 regulates the expression of NADPH:quinone oxidoreductase (NQO1) and hemeoxygenase-1 (HO-1) which participate in the antioxidant reactions shown in Figure 2. Unfortunately, there is no clear molecular mechanism in the CNS linking oxidative stress to a well-described innate mechanism of detoxification in which the mediators (e.g., glutathione, NADPH) and the source of reducing power (e.g., glucose) are known and placed in context. Indeed, a genomic approach has provided clear data on the complexity of looking into antioxidant mechanisms from the point of view of molecules involved, which are more than those originally thought [35]. Such report indicates: “*This work and other such studies may provide mechanisms for activating the ARE in the absence of general oxidative stress and a yet-unexploited therapeutic approach to degenerative diseases and aging*” [35]. In summary, whereas strengthening Nrf2 expression and its transcriptional activity is a good option to combat oxidative stress, the complementary option of enhancing the mediators and/or the source of “antioxidant” power is not yet explored. Remarkably, Nrf2 exists in various cell types of the brain and variation in the underlying mechanism in, say neurons *versus* astrocytes, may lead to specific strategies to combat CNS diseases. In other words, for any given disease coursing with neuronal death due to oxidative stress, it is not clear whether one should enhance the antioxidant potential of neurons or of (surrounding) glial cells [36,37,38,39]. Although it is reported that the Nrf2-ARE pathway is a good therapeutic target in neurodegenerative diseases [40,41], poor knowledge of underlying mechanisms leads to the appearance of controversies. One of them argues whether HO-1 protects neurons or induces neurodegeneration [42]. Finally, it should be noted that the glutathione-based mechanism also operates in brain (see [43,44,45,46] for review) but that its relevance in front of other brain innate mechanisms is not known. 

## 4. Upregulation of Nrf2 by Foods and/or Natural Compounds

Due to the growing interest in the Nrf2, studies have been undertaken to investigate ways to increase its concentration or its actions. Interestingly, some natural products have been tested with promising results. As reviewed in relationship with the benefits of some phytochemicals in regulating oxidative stress under a framework of chemoprevention to combat cancer, resveratrol and curcumin may upregulate Nrf2 [47]. Noteworthy, Toll-like receptors (TLR) are targeted by curcumin and dietary “anti-oxidant” phytochemicals inhibit TLR activation [48], thus impacting on inflammatory events such as those underlying neurodegenerative pathologies [49,50]. Rosemary extracts as well as some of its components, carnosol and carnosic acid, are also able to upregulate this transcription factor in cells from hepatic or neural lineage [51,52]. Ginseng has been also proposed to regulate Nrf2-ARE, hence having potential to combat neurological diseases [53]. Even assuming the relevance of supplements for strengthening antioxidant power via Nrf2 in peripheral tissues, the potential of the active compound(s) to enter the brain by crossing the blood brain barrier has not been, to our knowledge, tested. In this sense, to ascertain the real effectiveness of foods or supplements for increasing the neuroprotective potential of Nrf2 constitutes a challenge that is, unfortunately, difficult to address. Actually, one of the current challenges in CNS diseases related to age is how to determine the neuroprotective potential of a given intervention or a given drug. While drugs that improve symptoms exist, for instance in Parkinson’s disease, there is a shortage in preventive drugs and in neuroprotective drugs/interventions, being the lack of suitable neuroprotective markers (in humans) one of the main challenges to address neuroprotective power.
